# Mechanisms of Regulation of Cryptic Prophage-Encoded Gene Products in Escherichia coli

**DOI:** 10.1128/jb.00129-23

**Published:** 2023-07-13

**Authors:** Preethi T. Ragunathan, Evelyne Ng Kwan Lim, Xiangqian Ma, Eric Massé, Carin K. Vanderpool

**Affiliations:** a Department of Microbiology, University of Illinois Urbana-Champaign, Urbana, Illinois, USA; b Department of Biochemistry and Functional Genomics, University of Sherbrooke, Sherbrooke, Québec, Canada; Queen Mary University of London

**Keywords:** cryptic prophage, small protein, small RNA, antirepressor, lambda phage, RNA processing, lambdoid phage

## Abstract

The *dicBF* operon of Qin cryptic prophage in Escherichia coli K-12 encodes the small RNA (sRNA) DicF and small protein DicB, which regulate host cell division and are toxic when overexpressed. While new functions of DicB and DicF have been identified in recent years, the mechanisms controlling the expression of the *dicBF* operon have remained unclear. Transcription from *dicBp,* the major promoter of the *dicBF* operon, is repressed by DicA. In this study, we discovered that transcription of the *dicBF* operon and processing of the polycistronic mRNA is regulated by multiple mechanisms. DicF sRNA accumulates during stationary phase and is processed from the polycistronic *dicBF* mRNA by the action of both RNase III and RNase E. DicA-mediated transcriptional repression of *dicBp* can be relieved by an antirepressor protein, Rem, encoded on the Qin prophage. Ectopic production of Rem results in cell filamentation due to strong induction of the *dicBF* operon, and filamentation is mediated by DicF and DicB. Spontaneous derepression of *dicBp* occurs in a subpopulation of cells independent of the antirepressor. This phenomenon is reminiscent of the bistable switch of λ phage with DicA and DicC performing functions similar to those of CI and Cro, respectively. Additional experiments demonstrate stress-dependent induction of the *dicBF* operon. Collectively, our results illustrate that toxic genes carried on cryptic prophages are subject to layered mechanisms of control, some that are derived from the ancestral phage and some that are likely later adaptations.

**IMPORTANCE** Cryptic or defective prophages have lost genes necessary to excise from the bacterial chromosome and produce phage progeny. In recent years, studies have found that cryptic prophage gene products influence diverse aspects of bacterial host cell physiology. However, to obtain a complete understanding of the relationship between cryptic prophages and the host bacterium, identification of the environmental, host, or prophage-encoded factors that induce the expression of cryptic prophage genes is crucial. In this study, we examined the regulation of a cryptic prophage operon in Escherichia coli encoding a small RNA and a small protein that are involved in inhibiting bacterial cell division, altering host metabolism, and protecting the host bacterium from phage infections.

## INTRODUCTION

The establishment of lysogeny by temperate phages is a common occurrence in the environment, with nearly half of all sequenced bacterial genomes carrying prophages (1, 2). Whether a temperate phage will choose lysis or lysogeny depends on the metabolic state of the host cell and is driven by the activity of the phage repressor. Under specific conditions, the cognate repressor blocks transcription of the lytic genes and initiates the process of lysogeny, wherein the phage DNA is integrated into the host chromosome (1). In the absence of inducing signals, the repressor keeps a majority of phage genes transcriptionally silent in the bacterial lysogen (3). The integrated phage DNA, now called a prophage, stably replicates along with the host chromosome until a specific stress condition triggers the phage repressor to lose its control over the lytic genes, which initiates a cascade of events that drives the prophage to excise from the host chromosome and progress into the lytic cycle (4, 5).

The complex mechanisms involved in the regulation of prophage genes by the repressor and the physiological outcome of this regulation are illustrated by CI repression of λ prophage genes during lysogeny (6). CI and Cro are two repressors encoded in the immunity locus of λ. They are divergently transcribed and separated by an intergenic region that contains three operator sequences overlapping the respective promoters. For establishing and maintaining lysogeny, CI binds to two operator sequences closest to the *cro* promoter and blocks transcription of *cro*. Continuous repression by CI is necessary for stable maintenance of the prophage in the host cell. However, when the lysogen is exposed to inducing signals such as UV light or mitomycin C, CI becomes inactivated and *cro* gene expression begins. The newly synthesized Cro binds to the operators overlapping the *cI* promoter sequence and, in turn, blocks transcription of *cI*. With *cI* transcription blocked, the prophage genes necessary to initiate a lytic cycle are expressed and new λ progeny phages are produced (6–8). This genetic control of lytic and lysogenic cycles by two repressors is almost universally conserved in lambdoid phages, with some phages having additional layers of regulation (7).

Qin is one of the four lambdoid cryptic prophages of Escherichia coli K-12 and lacks the majority of the replication, head, and tail genes (9). Of particular interest in this prophage is the *dicBF* operon, which encodes the 53-nucleotide (nt) small RNA (sRNA) DicF and small (62-amino-acid) protein DicB. DicB interacts with MinC and targets it to the nascent septum at the cell center, resulting in MinC-dependent depolymerization of FtsZ and thus in cell division inhibition and filamentation of E. coli (10, 11). We recently showed that the small protein DicB confers an advantage on the host cell by providing superinfection immunity against certain phages. This protection is specific to phages that use the ManYZ inner membrane proteins of the mannose phosphotransferase system (PTS) to inject their DNA into the host cell (12). A second regulator in the *dicBF* operon, the sRNA DicF, base pairs with and inhibits *ftsZ* mRNA translation, limiting FtsZ protein synthesis (13). We found previously that DicF also directly affects host cell metabolism by inhibiting translation of pyruvate kinase, xylose regulator, and mannose transporter mRNAs (13, 14). These studies on DicB and DicF demonstrate how prophage-encoded regulators perform diverse functions, some of them beneficial, in the host cell.

The promoter of the *dicBF* operon, *dicBp*, is similar to the λ P_L_ promoter and is repressed by the DicA repressor (15, 16). The *dicAC* locus, located immediately upstream of the *dicBF* operon, is similar in arrangement and sequence to the immunity locus of lambdoid phages (16). Interestingly, DicA differs from the conventional lambdoid repressors in lacking the alanyl-glycyl bond necessary for RecA-mediated cleavage during the SOS response (16). The conditions leading to derepression of *dicBp* and production of DicB and DicF are still unknown.

In this study, we characterized the transcriptional and posttranscriptional mechanisms of regulation of the *dicBF* operon. During stationary phase, we observed that the sRNA DicF accumulates and is processed from the *dicBF* mRNA transcript by RNase III and RNase E. Characterization of transcriptional regulation of *dicBp*, the major promoter of the *dicBF* operon, revealed that DicA repression of *dicBp* is relieved by Rem, a putative antirepressor protein encoded on the Qin cryptic prophage. We show that the Rem antirepressor promotes filamentation of cells due to induced expression of the cell division inhibitors DicB and DicF. Our results also demonstrate that spontaneous induction of *dicBp* occurs in a subpopulation of E. coli cells when the *dicBF* operon is deleted and that stress conditions, including urea and high temperature, can also induce transcription from *dicBp*. Overall, this study identifies multiple distinct mechanisms by which the activity of an unconventional repressor of a lambdoid cryptic prophage is regulated and the consequences of derepression leading to the production of prophage-encoded products that influence various physiological processes of the host bacterium.

## RESULTS

### DicA represses transcription of the *dicBF* operon.

**FIG 1:**
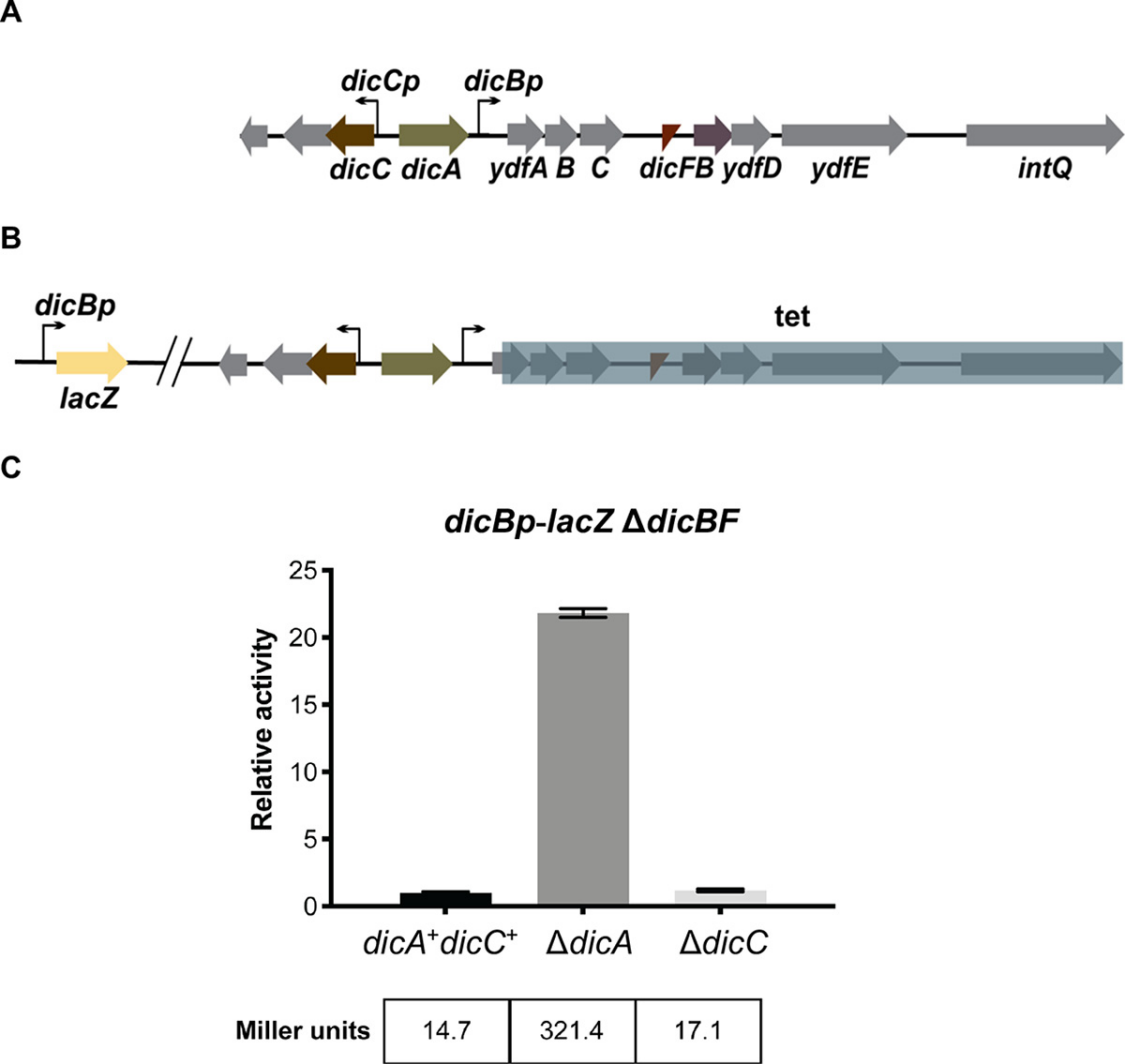
Regulation of the *dicBF* operon by DicA. (A) The *dicAC* locus is located immediately upstream of the *dicBF* operon and resembles the immunity locus of lambdoid phages. The promoter of the *dicBF* operon, *dicBp*, is repressed by DicA. (B) Reporter strain PR221 contains an out-of-locus *dicBp*-*lacZ* fusion, and the *dicBF* operon genes and *intQ* are deleted and replaced with a tetracycline resistance marker (*dicBp*-*lacZ* Δ*dicBF*). (C) β-Galactosidase activity of the *dicBp*-*lacZ* Δ*dicBF* strain was assayed with deletion of *dicA* and *dicC*. The specific activities in Miller units (indicated at the bottom) were normalized to the control strain (*dicA*^+^ *dicC*^+^) to obtain the relative activity for each experimental strain. Error bars show standard deviations from three biological replicates.

The *dicBF* operon of Qin cryptic prophage, which encodes the small RNA DicF and small protein DicB, is highly conserved in many strains of E. coli (13). The *dicBF* operon, which also includes *ydfA*, -*B*, -*C*, -*D*, and -*E*, is under the control of the regulators encoded by the *dicAC* locus, located immediately upstream of the operon (Fig. 1A) (16, 17). Apart from DicF and DicB, the only other characterized gene product of the *dicBF* operon is YdfD, which is a lysis protein (18). Previous studies have identified DicA as the repressor of the *dicBF* operon (16, 19, 20). The *dicAC* locus is similar to the immunity locus of lambdoid phages, with DicA similar to the CI repressor and DicC similar to Cro repressor (16). The intergenic regions between *dicA* and *dicC*, and *dicA* and *dicBp*, contain three operator sequences similar to those controlling λ P_R_ and P_L_ promoters, respectively (16, 20).

To confirm the roles of DicA and DicC in regulation of *dicBp* in our strain background, we constructed a transcriptional fusion with *dicBp* fused to *lacZ* placed at a locus distal to the Qin prophage (Fig. 1B). Next, we deleted the *dicBF* operon and *intQ* in this strain and replaced it with a tetracycline resistance marker (here called *dicBp*-*lacZ* Δ*dicBF*), which allows us to study the regulation of *dicBp* without the growth-inhibitory effect caused by induction of the *dicBF* genes (Fig. 1B) (13). We tested the roles of DicA and DicC in regulating *dicBp* by deleting the corresponding genes in the *dicBp*-*lacZ* transcriptional fusion strain and carrying out β-galactosidase assays (Fig. 1C). We found that deletion of *dicA* induced *dicBp* expression 22-fold compared to that with the control strain. Deletion of *dicC* did not affect *dicBp* activity (Fig. 1C). These results support the previous observations (16, 20) that DicA is the repressor of the *dicBF* operon.

### The sRNA DicF of the *dicBF* operon is expressed during stationary-phase growth.

The *dicBF* polycistronic transcript initiates upstream of *ydfA* and encompasses 6 protein-coding sequences (*ydfABC*, *dicB*, and *ydfDE*) and *dicF*, which codes for the DicF sRNA (Fig. 1A). Prior work suggested that DicF production requires processing of the longer polycistronic mRNA that initiates upstream of *ydfA* (17), but one study suggested that there is another promoter upstream of *dicB* (21). To understand the conditions and regulatory mechanisms governing expression of the *dicBF* operon, we first examined how DicF is produced.

**FIG 2:**
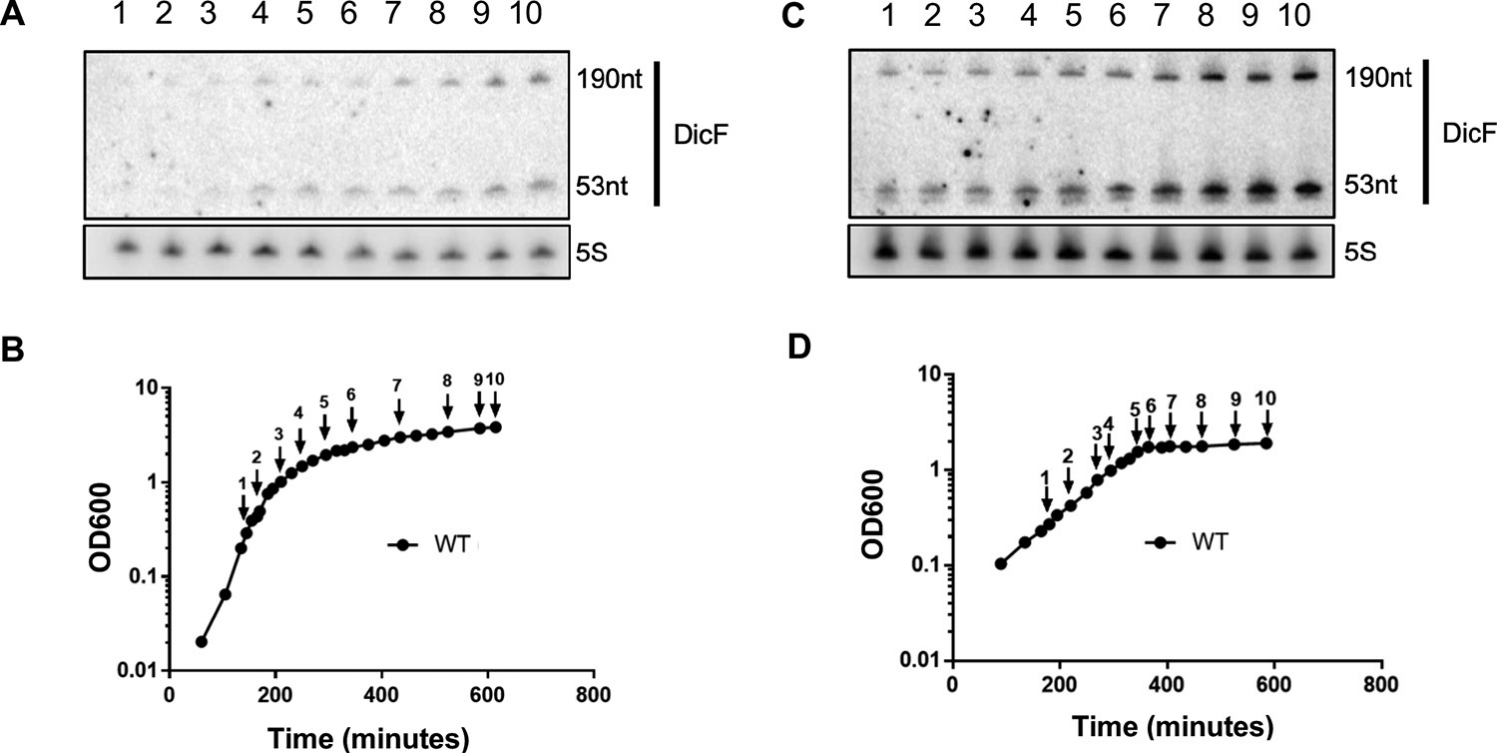
Production of the sRNA DicF. (A) Northern blots showing DicF levels in E. coli K-12 MG1655 grown in LB medium. 5S RNA was used as a loading control. (B) Growth curve of E. coli MG1655 in LB medium showing time points for RNA extraction. Numbers correspond to lanes in panel A. (C) Northern blots showing DicF levels in E. coli MG1655 grown in M63 minimal medium supplemented with 0.2% glucose and 1 μM FeSO_4_. 5S RNA was used as a loading control. (D) Growth curve of E. coli MG1655 in M63 minimal medium supplemented with 0.2% glucose and 1 μM FeSO_4_ showing time points for RNA extraction. Numbers correspond to lanes in panel C.

The sRNA DicF of the *dicBF* operon is one of the few cryptic prophage-encoded sRNAs whose relevance in the host bacterium has been established (13, 22, 23). To identify conditions that lead to the production of DicF in E. coli K-12, we tracked levels of this sRNA during growth in different media. RNA was extracted at various time points from wild-type (WT) E. coli K-12 MG1655 growing aerobically either in LB medium or M63 medium supplemented with glucose and iron (Fe). DicF levels were monitored by Northern blotting. Work by Faubladier et al. had shown that alternative processing of the DicF 5′ end by RNase III and RNase E generates a long (190-nt) species and a short (53-nt) species, respectively (17). Under our growth conditions, we were able to detect DicF fragments of both 190 nt and 53 nt in LB medium and M63 minimal medium supplemented with glucose and FeSO_4_ (Fig. 2A to D). Levels of both DicF species were low during log-phase growth but increased and remained stable during stationary phase in the two media used (Fig. 2A and B). Together, these results indicate that DicF is produced during stationary phase when E. coli is grown aerobically.

**FIG 3:**
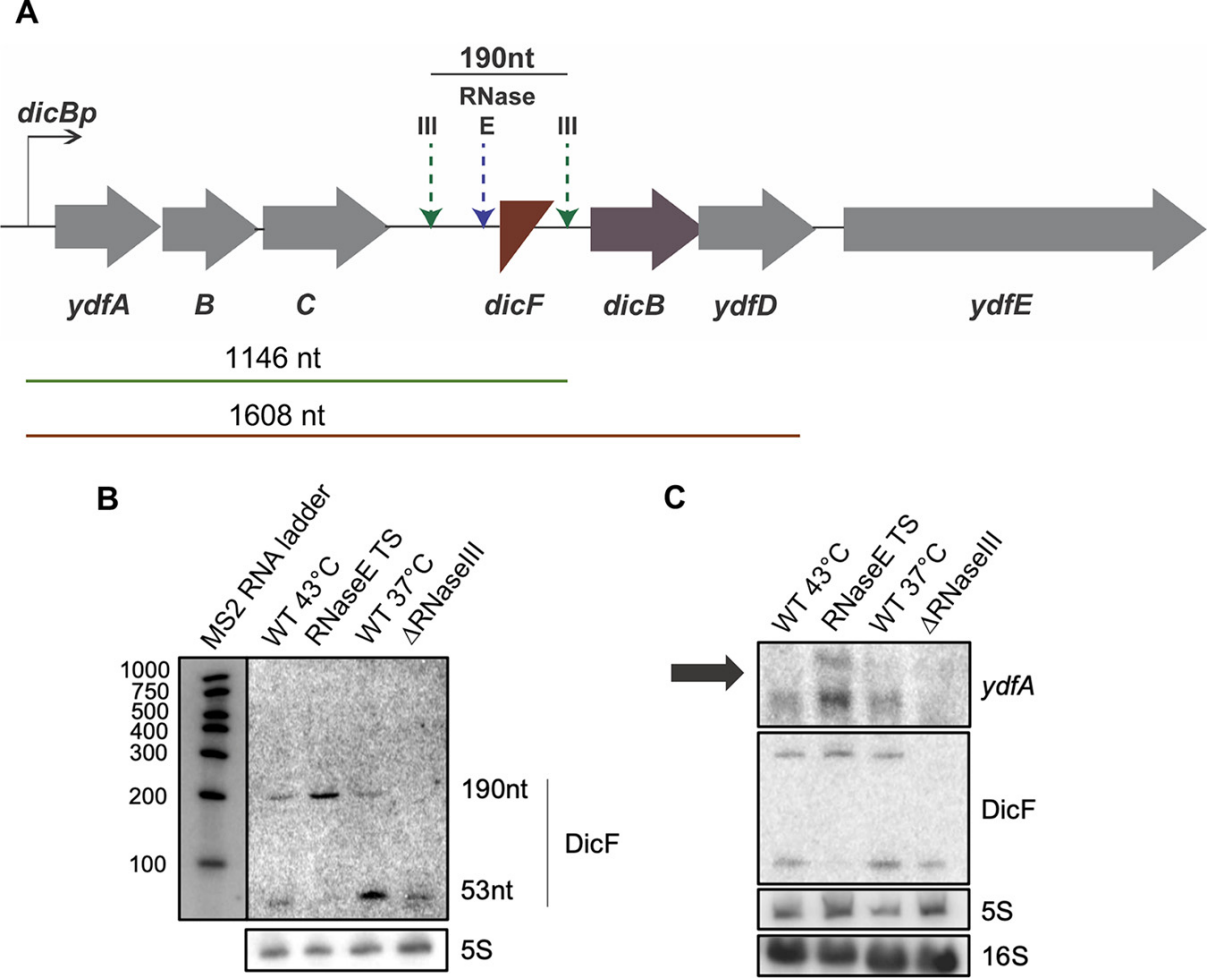
DicF is generated by processing of a transcript originating at *dicBp* by RNase III and RNase E. (A) RNase III and RNase E have been implicated in the processing of DicF from the *dicBF* transcript at the sites indicated (17). Transcripts that were generated from *dicBp* are noted below the operon illustration. (B) Northern blot showing DicF levels in strains grown in M63 minimal medium supplemented with 0.2% glucose and 1 μM FeSO_4_. RNase E (TS) is thermosensitive and was inactivated by heat shock at 43°C for 15 min. An MS2 ladder was used to determine each fragment’s length. 5S RNA was used as a loading control. (C) Northern blotting using probes against *ydfA* mRNA and DicF was performed using RNA extracted from overnight cultures of the indicated strains grown in M63 minimal medium supplemented with 0.2% glucose and 1 μM FeSO_4_. RNase E (TS) was inactivated as described for panel B. 5S and 16S RNAs were used as loading controls. The arrow indicates the location of the 1,541-nt 16S RNA.

Next, we investigated the mechanism by which the sRNA DicF was processed from the *dicBF* mRNA transcript during the stationary phase of E. coli growth. RNase III and RNase E have been implicated in previous studies (17) to yield the 5′ end of the functional DicF RNA from the longer transcript originating from *dicBp* (Fig. 3A). Using a strain with thermosensitive RNase E (TS) (24), we observed that the 190-nt DicF RNA fragment increased in abundance at 43°C, when RNase E (TS) was inactivated, while the 53-nt fragment was not detected (Fig. 3B). However, the 190-nt DicF fragment disappeared when RNase III was absent. Together, these data establish that a functional RNase E is required to generate the minimal 53-nt DicF fragment and RNase III cleavage is necessary for generating the longer 190-nt DicF fragment, supporting the previously suggested processing pattern of DicF (17). Primer extension analysis demonstrated that the 5′ end of the 190-nt DicF is generated by RNase III cleavage between positions 813 and 814 (see Fig. S1A and B in the supplemental material), consistent with previous observations (17) and suggesting that there is no additional promoter within the *dicBF* operon. Thus, both RNase III and RNase E play vital roles in transcript processing to generate the sRNA DicF.

We established that DicF was generated from the polycistronic transcript initiating at *dicBp* by probing for *ydfA* mRNA (Fig. 3C). We observed *ydfA* transcripts in cells grown to stationary phase, the same condition under which we observed the highest levels of DicF (Fig. 2D and 3C). The *ydfA* transcript was observed in WT cells at both 43°C and 37°C and was more abundant in the RNase E (TS) strain at the nonpermissive temperature (Fig. 3C), suggesting that RNase E-dependent processing upstream of *dicF* destabilizes the upstream region of the polycistronic transcript. Based on the location of the 16S RNA band in a control blot (1.5 kb, indicated by the arrow in Fig. 3C), we infer that the lower *ydfA* band observed was the 1,146-nt transcript encompassing *ydfABC*-*dicF* and the upper *ydfA* band, which accumulated only in the RNase E (TS) strain, corresponded to the 1,608-nt transcript of *ydfABC*-*dicFB*-*ydfD* (Fig. 3A and C). We did not detect any longer species that would correspond to *ydfABC-dicFB-ydfDE.* In the Δ*rnc* strain lacking RNase III, we did not detect either *ydfA* band, suggesting that RNase III-dependent processing may somehow stabilize the upstream portion of the polycistronic transcript. Finally, by performing reverse transcription-PCR (RT-PCR) on RNA extracted from WT cells grown in minimal medium, we demonstrated that transcripts encompassing the entire region between *ydfA-dicF* are detectable in WT cells (Fig. S2A and B). Together, these data strongly suggest that DicF is generated by RNase E- and RNase III-mediated processing from a polycistronic transcript originating from *dicBp* (upstream of *ydfA* [Fig. 3A]). Since we found no evidence for other promoters in this region, we proceeded to further study transcriptional regulation at *dicBp*.

### An antirepressor protein derepresses the *dicBF* operon.

DicA strongly represses *dicBp* (16) (Fig. 1C). DicA is predicted to bind to operator sequences that overlap the promoter and exclude RNA polymerase binding, similar to other lambdoid repressors (16). However, DicA differs from the conventional P22 or λ repressor because it is significantly shorter (135 amino acids, compared to 216 amino acids for P22 C2 repressor) and lacks the alanyl-glycyl bond that is necessary for RecA-mediated cleavage during the SOS response (16). This suggests that its activity may be regulated by a different mechanism than the conventional repressors. In a study by Lemire et al. (25), DicA was identified as one of the prophage repressors in E. coli that was similar in sequence to the Gifsy prophage repressors GfoR and GftR in Salmonella enterica serovar Typhimurium. These repressors also share other common features, such as having shorter length than the conventional lambdoid repressors and lacking the alanyl-glycyl bond. Instead, Gifsy repressor activity was found to be regulated by antirepressor proteins, encoded either on the same prophage or on another prophage harbored in the same strain (25). Due to the similarity of DicA to the Gifsy repressors, we sought to identify an antirepressor protein of DicA in E. coli K-12.

Using the protein sequence of GfoA, the antirepressor of Gifsy-1 prophage repressor, we performed a position-specific iterative BLAST (PSI-BLAST) search to find similar proteins in E. coli. Since many protein hits generated in the first round of PSI-BLAST did not have identifiable homologs in E. coli K-12, we performed a subsequent homology search using protein hits that had lengths comparable to that of GfoA. These secondary searches identified Rem, encoded by a gene on prophage Qin 3.6 kb upstream from the *dicBF* operon. Other potential antirepressors were identified on other cryptic prophages in the MG1655 genome. These included YpjJ of prophage CP4-57, YeeT of prophage Cp4-44, and YkfH of prophage CP4-6.

**FIG 4:**
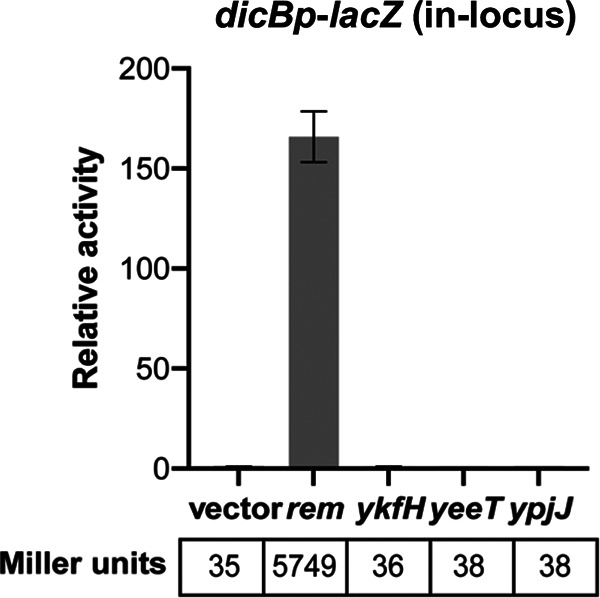
Rem acts as an antirepressor of *dicBp*. The predicted antirepressors were cloned on plasmids under P*_tet_* control and introduced to a strain carrying a *dicBp*-*lacZ* in-locus transcriptional fusion. The antirepressor genes were induced for 3 h with 10 ng/mL of anhydrous tetracycline, and β-galactosidase activity was assayed. The relative activity was calculated by dividing the Miller units of the specific strain by those of the vector control. Error bars show standard deviations from three biological replicates.

To test whether any of the putative antirepressors impacted induction of the *dicBF* operon, the genes encoding putative antirepressor proteins were placed under the control of an inducible (P*_tet_*) promoter and a heterologous ribosome binding site (RBS) for expression from a plasmid. These plasmids were introduced into a strain with an in-locus transcriptional *dicBp*-*lacZ* fusion to monitor transcription. Notably, because this is an in-locus fusion, the *lacZ* insertion effectively makes the fusion strain null for the cell division inhibitors DicB and DicF. Production of the predicted antirepressors was induced and Miller assays were carried out to quantify the β-galactosidase activity. Compared to the control strain, the strain expressing *rem* had a 165-fold increase in *dicBp* expression (Fig. 4). Strains producing the other putative antirepressors had *lacZ* activity similar to that of the control strain (Fig. 4). We confirmed that expression of *rem* from a construct with its native RBS also strongly induced the *dicBp* promoter (Fig. S3A). A third construct in which *rem* was placed under the control of a P*_lac_* promoter also resulted in derepression of *dicBp*-*lacZ* in a Δ*dicBF* strain (Fig. S3B). These results suggest that the cryptic prophage Qin encodes the Rem antirepressor that antagonizes the activity of DicA, resulting in derepression of *dicBp*.

### Rem induces filamentation of E. coli cells.

**FIG 5:**
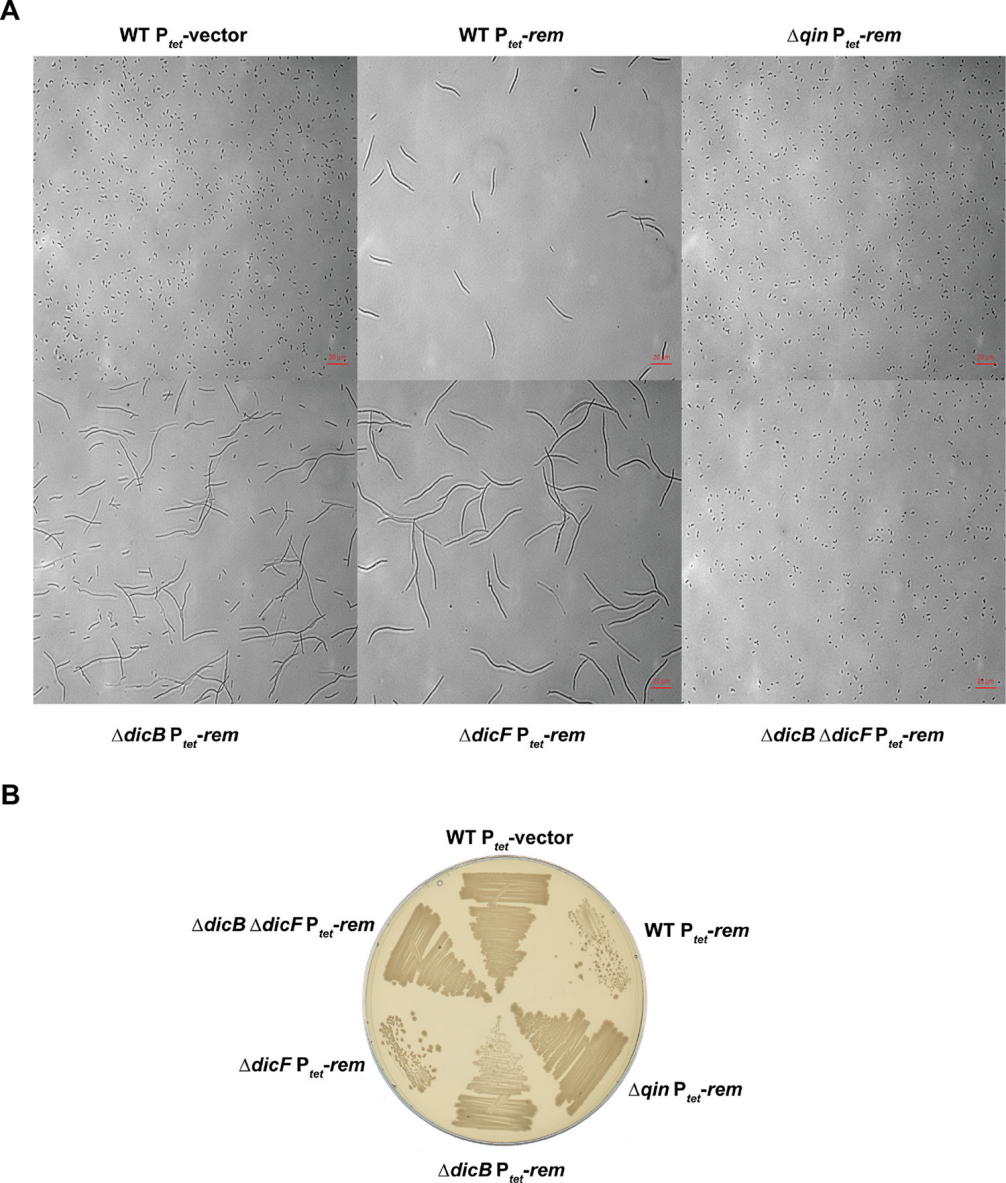
Expression of *rem* leads to filamentation and growth inhibition of cells. (A) WT strain and strains with deletions of *dicB*, *dicF*, *dicB dicF*, and the entire *qin* cryptic prophage harboring P*_tet_* vector or P*_tet_*-*rem* plasmids were grown for 3 h with 100 ng/mL of anhydrous tetracycline and observed under a microscope. (B) The strains used for panel A were streaked onto LB agar plates with 100 ng/mL of anhydrous tetracycline and incubated overnight at 37°C.

With the identification of Rem as the antirepressor of the *dicBF* operon, we wanted to examine Rem-dependent phenotypes and determine whether these were related to expression of the *dicBF* operon. Cells filament when the *dicBF* operon is expressed due to the combined activities of the cell division inhibitors DicB and DicF. For this experiment, *rem* was expressed in the WT strain, a *dicB* mutant, a *dicF* mutant, a *dicB dicF* double mutant, and a *qin* mutant (in which the entire Qin prophage was deleted). The WT strain expressing *rem* was highly filamentous compared to the same strain harboring the vector control (Fig. 5A). Expression of *rem* in the Δ*qin* background did not result in cell filamentation, suggesting that Qin-encoded gene products were responsible for the filamentous phenotype (Fig. 5A). Strains with individual deletions of *dicB* or *dicF* were filamentous when *rem* was expressed. However, in the Δ*dicB* Δ*dicF* strain, *rem* expression did not lead to filamentation, as cells appeared similar to the WT with the vector control. These results indicate that when *dicBp* is induced by a Rem-dependent mechanism, production of either DicB or DicF is sufficient to inhibit cell division. In strains expressing the other three putative antirepressors, we did not observe filamentation (Fig. S4A).

Growth of strains expressing *rem* yielded results consistent with the microscopy. The WT strain expressing *rem* was severely growth inhibited compared to the vector control strain, whereas in the Δ*qin* strain, Rem overproduction did not inhibit growth (Fig. 5B). The Δ*dicF rem*-expressing strain was more growth inhibited than the Δ*dicB rem*-expressing strain, consistent with our previous study indicating that the small protein DicB is a more potent growth inhibitor than the sRNA DicF (13). The Δ*dicB* Δ*dicF* strain was not growth inhibited and looked similar to the vector control strain (Fig. 5B). We did not observe growth inhibition when any of the other putative antirepressors were overproduced (Fig. S4B). Collectively, our results are consistent with the model that Rem acts as an antirepressor of the *dicBF* operon and expression of *rem* specifically derepresses *dicBp*, leading to production of the cell division inhibitors DicF and DicB.

We tested whether deletion of *rem* influences DicF levels during stationary-phase growth in minimal medium. Deletion of *rem* did not substantially affect accumulation of DicF (Fig. S5), suggesting that induction of the *dicBF* operon can occur by an antirepressor-independent mechanism.

### *dicBp* switches on spontaneously in a subpopulation of cells.

When an overnight culture of *dicBp*-*lacZ* Δ*dicBF* strain (Fig. 1B) was diluted and plated on LB agar plates with 5-bromo-4-chloro-3-indolyl-β-d-galactopyranoside (X-Gal; 40 μg/mL), we observed growth of a few blue colonies among a background of white colonies. The blue color indicates that these colonies induced *dicBp*-*lacZ* to a high level. This induction of *dicBp* in a subset of cells is reminiscent of the spontaneous induction of λ lysogens, which is a well-characterized phenomenon that occurs at low frequency (6, 26).

To further characterize the spontaneous induction phenomenon, *dicBp*-*lacZ* Δ*dicBF* strains that carried different deletions in *qin* prophage genes or the host factor *recA* were constructed (Table 1). We made dilutions of overnight cultures in phosphate-buffered saline (PBS) and plated reporter strains on LB agar plates with X-Gal. The frequency of colonies that showed high-level *dicBp* activity was quantified by counting the number of blue (promoter-on) and white (promoter-off) colonies. In the *dicBp*-*lacZ* strain with deletion of the *dicBF* locus, 1.16% of colonies were blue, demonstrating that ~1 out of every 100 cells in an overnight culture had switched on *dicBp* (Table 1).

**TABLE 1:** *dicBp* spontaneously induced in a subpopulation of cells^*a*^

Strain (*dicBp*-*lacZ*)	Genotype	% of *dicBp*-on colonies	SE
PR219	*dicBF* ^+^	0	0
PR221	Δ*dicBF*	1.16	0.55
PR231	Δ*dicBF* Δ*dicA*	100	0
PR232	Δ*dicBF* Δ*dicC*	0.04	0.07
XM252	Δ*dicBF* Δ*rem*	1.67	0.75
XM260	Δ*dicBF* Δ*recA*	1.02	0.54

a Overnight cultures of *dicBp*-*lacZ* strains were diluted in PBS and plated onto LB agar plates with X-Gal (40 μg/mL). The percentage of colonies with *dicBp* induced was calculated using the formula (number of blue colonies/total number of blue and white colonies) × 100. The standard error was calculated as the standard deviation of values from three biological replicates.

Deletion of *dicA* resulted in 100% blue colonies since the absence of DicA constitutively turns on *dicBp* (Table 1 and Fig. 1C). Deletion of the Cro-like *dicC* reduced the frequency of blue colonies to 0.04%. In phage λ, Cro repressor is responsible for flipping the bistable genetic switch by repressing *cI* transcription in a lysogen, which causes induction of lytic genes from P_L_ (6). In the absence of *cro*, P_L_ induction (due to derepression by CI) is not sustained, leading to lower levels of expression of lytic genes carried on the λ operon, which is analogous to the *dicBF* operon (6, 16). By analogy to the CI-Cro genetic switch, in strains lacking DicC, spontaneous induction of *dicBp* would not result in DicC-mediated repression of *dicA* transcription to reinforce the induction of *dicBF* operon transcription. This would result in restoration of DicA-mediated repression of *dicBp* and yield the lower frequency of cells that stably induce *dicBp* in the Δ*dicC* strain (Table 1). Together, these results suggest that DicA and DicC of the Qin cryptic prophage constitute a functional bistable genetic switch.

Spontaneous induction of the λ prophage occurs at a frequency of 1 in 10^5^ lysogens (27). SOS induction in a small population of cells was determined to play an important role in the spontaneous induction of λ lysogens, as a RecA deletion mutant and a CI mutant that does not get cleaved yielded reduced phage production from spontaneous induction (6, 28, 29). Furthermore, the Gifsy phage repressors, like DicA, do not directly respond to RecA-mediated cleavage but are still subject to RecA-mediated regulation indirectly through their antirepressors, which are under LexA control (25). Therefore, we wanted to determine whether RecA/SOS-mediated processes could impact spontaneous induction of *dicBp* by any mechanism. To check if this spontaneous induction of *dicBp* is mediated by RecA, we deleted *recA* in *dicBp*-*lacZ* Δ*dicBF* strain and found that the frequency of spontaneous induction was 1.02% (Table 1), similar to that for the *recA*^+^ strain. This implies that the observed *dicBp* derepression was independent of RecA activity. Next, we deleted the antirepressor *rem* in *dicBp*-*lacZ* Δ*dicBF* and quantified the frequency of *dicBp* induction. The frequency was 1.67%, which is similar to that of the *rem^+^* strain (Table 1). Thus, the observed mechanism of *dicBp* spontaneous induction is also independent of the antirepressor.

Interestingly, we did not observe any blue colonies with the *dicBF^+^ dicBp*-*lacZ* strain (Table 1). This is likely because high-level induction of *dicBp* would be toxic to the cells of this strain because both DicB and DicF are potent cell division inhibitors (13). Thus, *dicBF^+^* cells that induced *dicBp* in overnight culture would likely not form colonies. An alternative hypothesis is that a gene product encoded in the *dicBF* operon could contribute to maintaining tight DicA-mediated repression of *dicBp*.

### *dicBp* is induced by urea and high temperature.

A study with E. coli K-12 MG1655 showed that DicF-dependent filamentation occurred under anaerobic conditions, due to increased stability of DicF under anaerobic conditions and faster degradation under aerobic conditions (30). Another study showed that under microaerobic conditions, four DicF orthologs encoded by different prophages in E. coli O157:H7 are produced (22). However, we have not observed DicB or DicF-mediated filamentation of MG1655 cells under anaerobic conditions or increased expression of DicF under microaerobic conditions (data not shown). We also tested the expression of *dicBp*-*lacZ* in response to the SOS-inducing agent mitomycin C by disk diffusion assay on indicator plates and observed that unlike λ and other lambdoid prophages, mitomycin C does not induce *dicBp* (data not shown).

**FIG 6:**
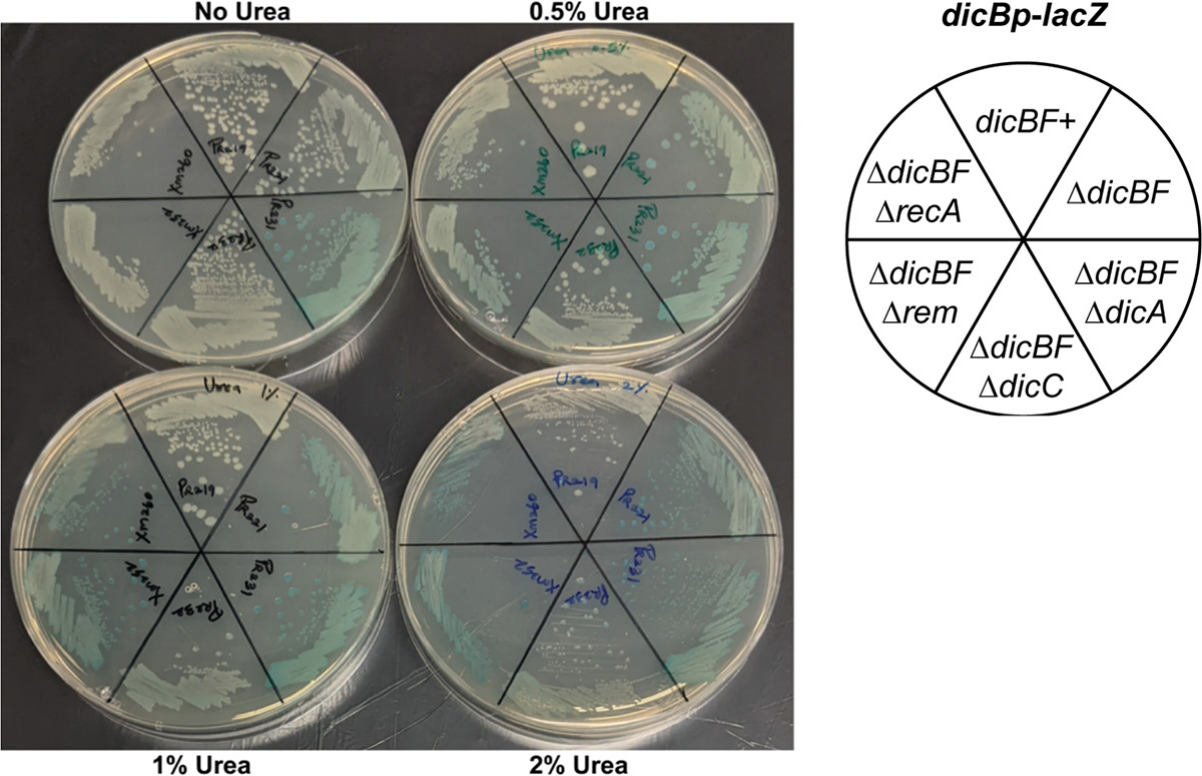
*dicBp* is induced by urea. *dicBp*-*lacZ* strains with different deletions of the *dic* locus, *rem*, and *recA* were streaked onto LB agar plates with X-Gal (40 μg/mL) and urea at the indicated concentrations and incubated overnight at 37°C.

To identify other conditions that induce expression of the *dicBF* operon, we carried out a screen using Biolog plates (31). We grew the *dicBp*-*lacZ* Δ*dicBF* strain in 14 different 96-well Biolog phenotype microarray plates. Plates contained different carbon sources, antibiotics and other chemicals, or compounds that induced different osmotic and ionic effects and pH changes. X-Gal was added to plates to monitor LacZ activity. These assays identified urea as an inducer of *dicBp-lacZ*. To further characterize induction of *dicBp* by urea, we streaked Δ*dicBF* strains harboring *dicBp*-*lacZ* on LB X-Gal plates with different concentrations of urea (Fig. 6). We observed that *dicBp*-*lacZ* Δ*dicBF* colonies were white on plates with no urea but turned blue when urea was present, with colonies turning darker blue in increasing concentrations of urea up to 2% (Fig. 6). The *dicBp-lacZ* Δ*dicBF* Δ*rem* and *dicBp-lacZ ΔdicBF* Δ*recA* strains were induced to levels similar to those the *dicBp-lacZ ΔdicBF* parent strain, indicating that *dicBp* induction by urea was independent of the antirepressor Rem and the SOS response (requiring RecA) (Fig. 6). The *dicBp-lacZ ΔdicBF* Δ*dicA* strain was blue with and without urea, as expected since it lacks the repressor. We observed that *dicBp-lacZ ΔdicBF* Δ*dicC* colonies turned only a light blue color on 2% urea plates and were not as highly induced as the *dicBp-lacZ ΔdicBF* parent (Fig. 6). This suggests that the induction of *dicBp* by urea could occur via flipping of the bistable switch between DicA-mediated repression of *dicBp* to DicC-mediated repression of *dicA*, as seen previously with the spontaneous-induction phenotype. Finally, we did not observe *dicBp-lacZ dicBF^+^* colonies turning blue in the presence of urea.

**FIG 7:**
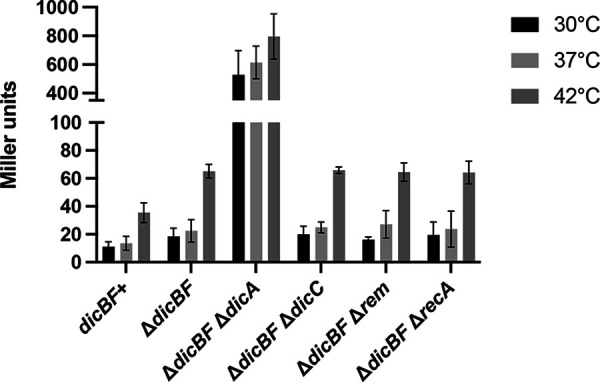
*dicBp* is induced by high temperature. The indicated strains were cultured at 30, 37, or 42°C from overnight cultures grown at 30°C. When the strains reached an OD_600_ of ~0.8, β-galactosidase activity was assayed. Error bars show standard deviations from three biological replicates.

We hypothesized that induction of *dicBp* by urea might be due to DicA instability because urea is a protein-denaturing agent. Since high temperature is another physiological condition that destabilizes proteins, we tested *dicBp* expression in our reporter strains at different temperatures. At 30°C and 37°C, all strains except the *dicBp*-*lacZ* Δ*dicBF* Δ*dicA* strain were white on LB X-Gal plates (Fig. S6). At 39°C, the *dicBp*-*lacZ* Δ*dicBF*, *dicBp*-*lacZ* Δ*dicBF* Δ*rem*, and *dicBp*-*lacZ* Δ*dicBF* Δ*recA* strains turned light blue. At 42°C, the colonies of these strains turned a darker blue (Fig. S6). This shows that DicA-mediated repression of *dicBp* is relieved at 39 and 42°C in strains lacking *dicBF*, and this effect was independent of Rem and RecA. As observed with urea, the *dicBp*-*lacZ* Δ*dicBF* Δ*dicC* strain turned only a light blue at 42°C (Fig. S6). The *dicBp*-*lacZ dicBF*^+^ colonies turned a very light blue only at 42°C (Fig. S6). In β-galactosidase assays performed using liquid cultures, all strains except the *dicBp*-*lacZ* Δ*dicBF* Δ*dicA* strain had very low β-galactosidase activity at 30°C and 37°C (Fig. 7). The β-galactosidase activity of the *dicBp*-*lacZ* Δ*dicBF* Δ*dicA* strain remained high at all the three temperatures tested since the repressor was deleted in this strain. At 42°C, the β-galactosidase activity of the remaining strains increased 3- to 4-fold compared to the activity of the same strain at 30°C (Fig. 7). It was interesting that the *dicBp*-*lacZ dicBF*^+^ and *dicBp*-*lacZ* Δ*dicBF* Δ*dicC* strains also had a similar increase in β-galactosidase activity at 42°C compared to the respective strains at 30°C, which was somewhat different than what we observed on the X-Gal plates (Fig. 7 and Fig. S6). Another important observation was that the β-galactosidase activity of the *dicBp*-*lacZ* Δ*dicBF* strain was approximately twice that of the *dicBp*-*lacZ dicBF*^+^ strain at all three temperatures (Fig. 7). Overall, this suggests that *dicBp* is derepressed at higher temperature and the presence of the wild-type *dicBF* operon contributes to the repressive effect on *dicBp*.

## DISCUSSION

**FIG 8:**
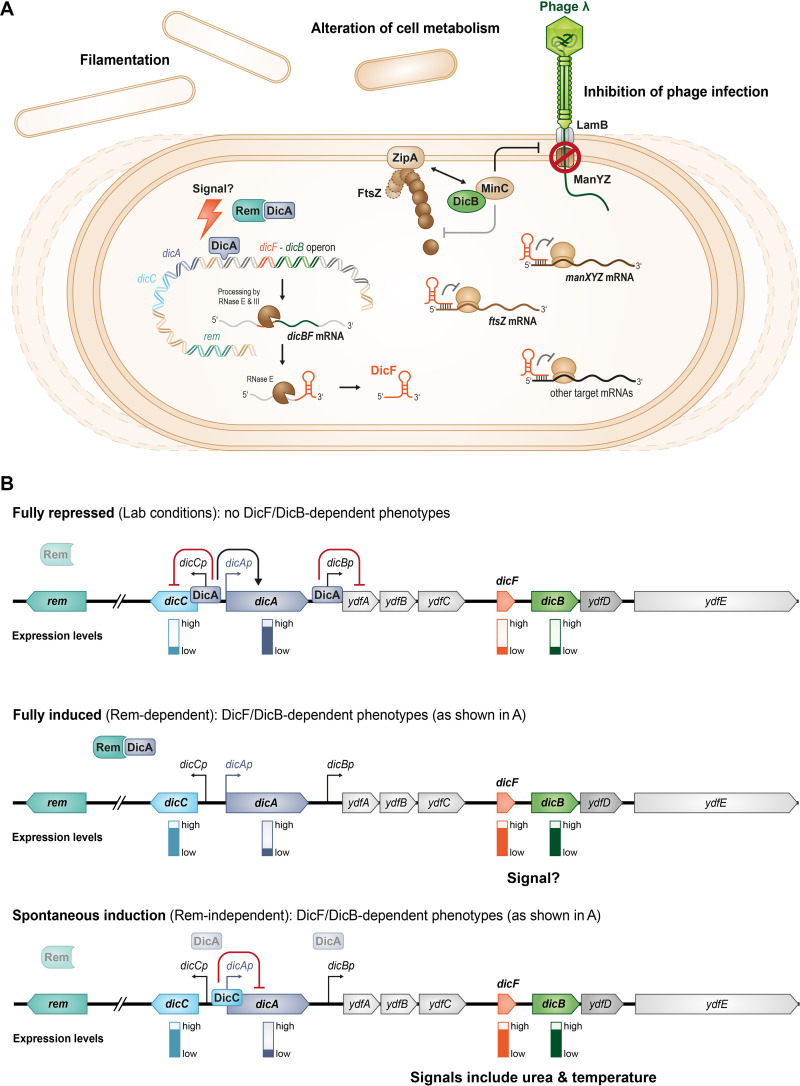
Working model for regulation of the *dicBF* operon. (A) The sRNA DicF and the small protein DicB are encoded on the Qin prophage of E. coli K-12. In this study, we showed that DicF accumulates during stationary phase and is generated from the polycistronic mRNA by RNase E- and RNase III-dependent processing. Previous work demonstrated that DicF base pairs with and prevents translation of *ftsZ* and *manXYZ* mRNAs, resulting in inhibition of cell division and alteration of carbohydrate metabolism, respectively. DicB affects FtsZ and ManXYZ protein activity. DicB localizes MinC, a negative regulator of FtsZ polymerization, to the septum to inhibit cell division. The DicB-MinC complex also inhibits ManXYZ function and prevents transport of mannose and infection by phages that require ManXYZ for DNA injection. (B) The *dicBF* operon is regulated by DicA and DicC, which are analogous to λ phage CI and Cro, respectively, and encoded upstream of *dicBp*. DicA represses transcription of the *dicBF* operon and *dicC*. Transcription at *dicBp* is regulated by several mechanisms. The antirepressor Rem eliminates the repressive effect of DicA on *dicBp* and induces production of DicF and DicB, resulting in filamentation. *dicBp* can also be induced spontaneously in a subset of cells. DicC is important for this phenotype, which resembles the λ bistable switch. Environmental signals like urea and high temperature can impact the bistable switch and induce transcription from *dicBp* in a DicC-dependent manner.

The roles of the cryptic prophage products DicB and DicF in protecting host cells from phage infections (12), altering host metabolism (13), and inhibiting cell division (10, 13, 15, 32) indicate the complex relationship that exists between the host cell and the Qin cryptic prophage (Fig. 8A). The evolutionary maintenance of these regulators hints at possible fitness advantages that DicB and DicF confer on the host cell under specific conditions. Protection of host cells from phage infections by DicB is a clear example of such a beneficial relationship (12). In this study, we investigated the mechanisms by which production of DicB and DicF are regulated and found several levels of control that appear to modulate the stability or activity of the prophage repressor protein DicA (Fig. 8A and B). DicA represses the *dicBF* operon and *dicC*, resulting in low levels of expression of DicB and DicF (Fig. 1C) (16, 19). Nevertheless, we were able to detect expression of the *dicBF* operon from *dicBp* leading to production of DicF via RNase E- and RNase III-dependent processing of the *dicBF* polycistronic mRNA (Fig. 3) (17). DicF accumulates particularly in stationary phase (Fig. 2). We identified an induced state wherein DicA-mediated repression of the *dicBF* operon is abrogated by an antirepressor protein Rem (Fig. 8B), which is encoded on the same cryptic prophage (Fig. 4). Ectopic production of Rem leads to derepression of the *dicBF* operon, resulting in production of DicB and DicF and causing cell filamentation (Fig. 4 and 5). We do not yet know what signal stimulates the Rem-dependent fully induced state. Production of DicF during the stationary phase of growth is independent of Rem (Fig. S5), suggesting that there is at least one additional mechanism for induction of the *dicBF* operon. Spontaneous induction of *dicBp* in a subpopulation (~1%) of cells is also independent of the Rem antirepressor protein (Fig. 8B and Table 1). The spontaneous induction appears to occur by a mechanism that is reminiscent of the phage λ bistable switch. We showed that transcription of the *dicBF* operon can be induced by external factors like urea and high temperature via a Rem-independent mechanism which also influences the bistable switch of DicA and DicC (Fig. 6 and 7). Collectively, we uncovered multiple conditions and mechanisms that impact expression of the *dicBF* operon of the Qin cryptic prophage.

The induction of a functional prophage in a lysogen is primarily dependent on repressor inactivation. Representatives of a major class of repressors, like the λ prophage repressor CI, get directly cleaved during the SOS response, which results in expression of the lytic genes that were under its control (6). However, there is another class of repressors that do not get cleaved during the SOS response. The Gifsy prophages of Salmonella encode the repressors GfoR and GfhR, which fall into this category (25). The regulation of these repressors involves antirepressor proteins that directly interact with and disassociate the cognate repressor from the operator sequence, resulting in transcription from the derepressed promoter (25). These antirepressors are under the control of LexA, which responds to SOS-inducing conditions (25). It is interesting that a cryptic prophage like Qin has an intact regulation module consisting of the repressor and the antirepressor. Given the multiple functions of DicB and DicF in the host cell, and the conservation of the *dicBF* operon in many E. coli strains (12, 13), expression of the *dicBF* operon could be beneficial for the host under specific conditions. Unlike for the Gifsy antirepressors, the condition that induces antirepressor Rem production is not known. Remarkably, a functional temperate phage, named mEp460_evo81 (33), was recently isolated from the virome of infant feces, and it harbors genes similar to *dicB*, *dicF*, *dicAC*, and *rem*. Our analyses indicate that DicA and Rem of mEp460_evo81 share similarity with E. coli K-12 DicA and Rem in size and amino acid sequence. The conservation of DicA and Rem in a functional phage suggests that the regulation of DicA by the antirepressor Rem is important for the life cycle of the phage and that signals that are specific to the environment that the host bacterium resides in could trigger derepression of the *dicBF* operon through Rem.

A recent study suggested that one additional mechanism of regulation of the *dicBF* operon involves repression by the nucleoid-associated protein (NAP) Fis and the RNA chaperone protein Hfq. Fis and Hfq, along with other known NAPs, were found to occupy extended domains of the E. coli K-12 chromosome, including cryptic prophages, and silence transcription of genes located in these domains (34). Combining mutations in *hfq* and *fis* was lethal in E. coli K-12, but growth was partially rescued by the deletion of either of the cryptic prophages Qin and DLP-12 (34). Both Qin and DLP-12 contain intact phage lysis cassettes that can lyse the host cell when expressed. Additionally, Qin harbors DicB, DicF, and HokD, three gene products whose prolonged expression can directly affect bacterial growth and survival (13, 35). We speculate that Hfq and Fis could be involved in regulating the immunity regions of these cryptic prophages to silence prophage genes that can be toxic to the host cell when overexpressed.

The bistable switch of λ controls complex genetic circuits involved in the decision between the lysogenic or lytic life cycle. CI and Cro are the two repressors involved in this genetic switch. The role of CI is to block expression of genes involved in the lytic cycle by binding to the operator sequences at P_R_ (analogous to *dicCp*) and P_L_ (analogous to *dicBp*) (Fig. 8B) (6). CI binding to P_R_ prevents transcription of *cro*, while also activating its own transcription. Under stress conditions that lead to inactivation of CI, Cro is produced, which in turn blocks *cI* expression. This depletion of CI leads to the expression of λ lytic genes and the prophage commits to the lytic cycle (6, 36). DicA and DicC are similar to λ CI and Cro in terms of their structural similarity and binding to operator sequences in the *dicCp* (P_R_) and *dicBp* (P_L_) promoters (16). A study by Yun et al. in E. coli MG1655 showed that by binding to *dicCp*, DicA blocks *dicC* transcription and activates its own transcription (20). Recent work in E. coli O157:H7 showed that overexpression of DicC repressed *dicA*’s transcription, similar to Cro repression of *cI* (37). Additionally, deletion of *dicC* led to a reduction in *dicB* levels while overexpression induced *dicB* expression (37). Our results show that DicC is important for the spontaneous-induction phenotype of *dicBp*, as deletion of *dicC* results in a reduction in the frequency of cells that have induced *dicBp*. DicC is also necessary for a strong induction of *dicBp* with urea and high temperature. Thus, along with supporting data from previous studies, our results are consistent with a model in which the cryptic prophage repressors DicA and DicC form a functional bistable switch in E. coli K-12, and an inherent instability of DicA causes derepression of *dicBp* and initiates the lytic cycle equivalent of Qin in a subpopulation of cells (Fig. 8B).

The cryptic prophages of the model bacterium E. coli K-12 were shown to increase resistance of the host to environmental stresses, like oxidative stress and osmotic stress, and to certain antibiotics, like quinolones and β-lactams (38). Since prophages also encode gene products, like lytic proteins and toxins, that can have undesired effects on the host cell viability, it is common for such prophage genes to be strongly repressed in the host bacterium. So far, very few studies have investigated the mechanisms that lead to derepression of the repressors of E. coli K-12 cryptic prophages, especially those of unconventional repressors like DicA. The E. coli K-12 cryptic prophage Rac also encodes an unconventional repressor, called RacR, that shares similarity with the Gifsy prophage repressors and does not respond to SOS response (25). Remarkably, Rac also encodes a Cro-like protein called YdaS (4), suggesting that the bistable switch of λ phage CI-Cro could exist in yet another cryptic prophage of E. coli K-12. Based on the findings from the current study, it would be interesting to explore if cryptic prophages with DicA-like unconventional repressors in E. coli K-12 and other bacteria encode the associated antirepressors to regulate the expression of genes under repressor control. Exploring the relationship between the conditions that lead to the expression of cryptic prophage genes and functions of the associated gene products in the host will provide further understanding on the complex role of cryptic prophages in host bacteria.

## MATERIALS AND METHODS

### Strain construction.

All the strains and phages used in this study are summarized in Table S1, and the oligonucleotides (from Integrated DNA Technologies) are listed in Table S2. The strains used in the study are derivatives of E. coli K-12 strain MG1655. Chromosomal mutations were constructed using the λ red recombination method as described previously (39–41).

The in-locus *lacZ* fusion to *dicBp* was constructed as described previously (42). Briefly, the primers O-PR124 and O-PR125, which contain 40 nt of sequence homologous upstream and downstream of *dicBp* and 20 nt of sequence homologous to the FLP recombination target (FRT)-chloramphenicol cassette, were amplified using pKD3 as the template. This DNA fragment was recombined into the bacterial chromosome at the *dicBp* site using λ red recombination. Using PCP20, the chloramphenicol cassette was flipped out and *lacZ* was inserted using pKG136 plasmid (derivative of pCE36). Using P1*vir*, the in-locus *dicBp*-*lacZ* fusion was moved into DB166 to generate PR136.

The out-of-locus *dicBp* transcriptional fusion carried in strain PR219 was first constructed in PM1805 (43) by λ red recombination of a DNA fragment generated by amplification using primers O-PR241 and O-PR242 with WT DNA as the template. This fragment contained *dicBp* and homologies to the region upstream of P_BAD_ and *lacZ*, such that P_BAD_ was replaced upon recombination. The *ydfA*-*intQ*::tet deletion was constructed by amplification using primers O-PR230 and O-PR231 and genomic DNA from strain DB181 as the template to amplify the tetracycline resistance marker and recombined into the bacterial chromosome. Using P1*vir*, this deletion was moved into PR219, generating strain PR221. Strains PR226 (O-PR299/O-PR300), PR227 (O-PR303/O-PR304), XM249 (O-PR307/O-PR308), and PR221 Δ*recA*::cm (O-PR311/O-PR312) were constructed using the indicated primers (names in parentheses) with pKD3 as a template and recombined into PR221 pSIM6 by λ red recombination. The chloramphenicol cassette in PR226, PR227, XM249, and PR221 Δ*recA*::cm was removed using pCP20, leaving an FRT scar in place and generating strains PR231, PR232, XM252, and XM260, respectively.

The Δ*qin*::kan, Δ*dicB*::kan, and Δ*dicF*::kan deletions were moved from DB237, DB241, and DB252 (13) into DB166 using P1*vir* transduction to generate PR142, PR143, and PR144, respectively. Using pCP20, the kanamycin marker was removed, leaving an FRT scar and resulting in strains PR145, PR146, and PR147. A Δ*dicB*::kan mutation was constructed using primers O-DB508 and O-DB509 (13) with pKD13 as the DNA template and recombined into PR147 pSIM6 to generate PR148. Using pCP20, DB166 Δ*dicF* Δ*dicB* was generated from PR148 and named PR149.

A Δ*dicF*::kan allele was constructed using primers EM2782 and EM2783 with pKD4 as the template. The resulting product was recombined in strain EM1055 (44) pKD46 to generate ENG63. Similarly, a Δ*rem*::cat deletion was constructed using primers O-PR307 and O-PR308 and recombined into EM1055 pKD46, generating ENG262.

Oligonucleotides containing NdeI and BamHI restriction sites were used to amplify the predicted antirepressor coding sequences from the chromosome of E. coli K-12. The vector pZA31R (45) and PCR products were digested with NdeI and BamHI restriction enzymes and ligated using DNA ligase to generate plasmids pZAPR2, pZAPR3, pZAPR4, and pZAPR5 with the antirepressor genes under P_LtetO-1_ control. The antirepressor-encoding *rem* was also cloned into pZA31R using oligonucleotides that preserved its native RBS, yielding plasmid pZAPR6. DicF was cloned into pZA31R by amplification with primers O-PR147 and O-PR148, which contained the NdeI and BamHI sites, yielding pZAPR1. The vector pBRCS12 and PCR products were digested by BamHI and HindIII restriction enzymes and ligated to generate plasmid pBRPR7 with *rem* under P*_lac_* control.

### Bioinformatic prediction of antirepressors of DicA.

Using the protein sequence of GfoA, the antirepressor of Gifsy-1 prophage repressor (25), PSI-BLAST search was performed to find similar proteins in E. coli. Since many protein hits generated in the first round of PSI BLAST did not have identifiable homologs in E. coli K-12, a subsequent homology search was performed using protein hits that had lengths comparable to those of GfoA. When the protein WP_171885951.1, generated from round one of PSI-BLAST, was used as a query, Rem encoded by Qin prophage was identified with 33% identity. Using a similar procedure with FsoA, the antirepressor of Fels-1 prophage (25), Rem was identified again as a possible candidate. By altering the BLASTp parameters, proteins YpjJ of prophage CP4-57, YeeT of prophage Cp4-44, and YkfH of prophage CP4-6 were also identified as other potential antirepressors in E. coli K-12 when FsoA was used as a query.

### β-Galactosidase assay.

Strains were diluted 1:100 from overnight cultures in Tryptone Broth (TB) medium and grown to mid-logarithmic phase at 37°C on a rotary shaker. TB medium was supplemented with 100 μg/mL of ampicillin for pBRCS12 derivative plasmids, and 0.1 mM isopropyl-β-d-thiogalactopyranoside (IPTG) was added to induce expression of genes under P*_lac_* control for 1 h, after cells reached an optical density (OD) of 0.2 to 0.3. A total of 25 μg/mL of chloramphenicol was added to TB medium when pZA31R-derived plasmids were used, and 10 ng/mL of anhydrous tetracycline was added to induce expression of genes under P*_tet_* control for 3 h. After cultures reached the mid-logarithmic phase, β-galactosidase activity was quantified as described previously using Miller assays (46).

For experiments in which β-galactosidase activity was assayed at different temperatures, strains were grown overnight at 30°C in TB medium and subcultured 1:100 into TB medium. The strains were grown at three different temperatures, 30°C, 37°C, and 42°C. When cultures reached an OD at 600 nm (OD_600_) of ~0.8, 500 μL of sample was taken from each culture and β-galactosidase activity was quantified.

### Microscopy.

Strains harboring the indicated plasmids were subcultured 1:100 in LB medium with 25 μg/mL of chloramphenicol and 100 ng/mL of anhydrous tetracycline and grown in a rotary shaker for 3 h at 37°C. After 3 h, the cultures were transferred to ice. Five-microliter volumes of samples were placed on a 24- by 50-mm no. 1.5 coverslip, and a 1.5% agarose gel pad was placed on the cells for immobilization. Cells were then imaged using a Zeiss apotome microscope under bright-field setting and imaged at a magnification of ×40.

### Growth assays.

Strains harboring the indicated plasmids were streaked on LB plates with 100 ng/mL of anhydrous tetracycline to induce expression of genes under P*_tet_* control, and the plates were incubated overnight at 37°C.

### Dilution plating assay.

Overnight cultures of the indicated strains in Fig. 6 were serially diluted in 1× PBS. A total of 50 μL of the 10^−5^ dilution was plated on seven LB plates with X-Gal for each strain to obtain countable colonies. The plates were incubated overnight at 37°C. The next day, the blue and white colonies were counted. For each biological replicate, a total of 800 to 1,600 colonies were counted from seven plates for each strain. The frequency of blue colonies was calculated using the formula (number of blue colonies/total number of blue and white colonies) × 100.

### Biolog assays.

The Biolog experiment was conducted according to the protocol indicated previously (31), with a key variation of adding X-Gal at a concentration of 1.25 mM to IF-0 and IF-10 growth media, instead of the dye mix. Briefly, an overnight culture of PR221 grown in LB medium was centrifuged to remove the LB medium and the pellet was resuspended in IF-0 medium. This was further diluted in IF-0 and IF-10 media supplemented with 1.25 mM X-Gal to attain the prescribed OD values stated previously (31). A total of 100 μL/well was aliquoted into plates PM1, -2, and -9 to -20, and the plates were incubated overnight at 37°C. Plates PM1 and -2 contained different carbon sources, plate PM9 contained osmolytes, plate PM10 contained wells of different pHs, and plates PM11 to -20 contained different chemicals. On the next day, the wells were scored based on the presence of a dark blue color.

### Growth on urea and different temperatures.

LB agar plates with X-Gal (40 μg/mL) or X-Gal and urea at final concentrations of 0.5%, 1%, and 2% were prepared. A single colony for each of the indicated strains was picked and streaked, and the plates were incubated overnight at 37°C. Similarly, the strains were streaked on four LB agar plates with only X-Gal (40 μg/mL) and incubated at 30, 37, 39, and 42°C overnight.

### RNA extraction and Northern blotting.

Strains were diluted 1:1,000 in LB medium or adjusted to an OD of 2 in M63 minimal medium supplemented with 0.2% glucose and 1 μM FeSO_4_ and grown in a rotary shaker. RNA samples were extracted using the classic hot-phenol protocol as described previously (47) at the indicated times or ODs. A total of 5 to 10 μg of total RNA was loaded onto a 5 to 10% acrylamide gel containing 8 M urea. Electrotransfer was done on Hybond-XL membranes for 1 h at 200 mA, with cross-linking under 254-nm UV for 45 s. Membranes were prehybridized in Church buffer (48) for 1 h at 42°C, and radiolabeled DNA or RNA probes detailed in Table S2 were added overnight. Membranes were washed, then exposed to phosphor screens, and revealed with GE Healthcare Typhoon Trio.

### Primer extension assays.

Strains were diluted 1:1,000 in LB medium grown in a rotary shaker. RNA samples were extracted using the classic hot-phenol chloroform protocol as described previously (47) after cells reached an OD of 0.5 to 0.6. Primer extension was then performed following the protocol previously described (49). A total of 20 μg of total RNA was used with radiolabeled primer EM4753 to generate cDNA that was migrated on an 8% acrylamide gel containing 8 M urea. The sequencing ladder was generated by PCR with radiolabeled probe EM4753 from a DNA matrix using primers EM2784 and EM4668. The gel was exposed on a phosphor screen and revealed with GE Healthcare Typhoon Trio.

### RT-PCR assays.

Cultures were adjusted to an OD of 2 in M63 minimal medium supplemented with 0.2% glucose and 1 μM FeSO_4_ and grown in a rotary shaker. RNA samples were extracted using the classic hot-phenol protocol as described previously (47) after cells reached an OD of 1.8. RNA extracts were treated with TURBO DNase (Thermo Fisher Scientific), and reverse transcription was performed with primer EM4722, Protoscript II (New England BioLabs [NEB]), and 0.1 M dithiothreitol (DTT). PCR was then performed on cDNA and strain EM1055 with *Taq* enzyme and primers listed in Table S2.
