# Supplementary material for: Mechanisms of Regulation of Cryptic Prophage-Encoded Gene Products in Escherichia coli
Source: J Bacteriol. 2023 Jul 13;205(8):e00129-23. doi: 10.1128/jb.00129-23 (PMC10448788; doi:10.1128/jb.00129-23)
Supplement: Supplemental file 1 — Fig. S1 to S6 and Tables S1 and S2. Download jb.00129-23-s0001.pdf, PDF file, 10.1 MB [file jb.00129-23-s0001.pdf]

A

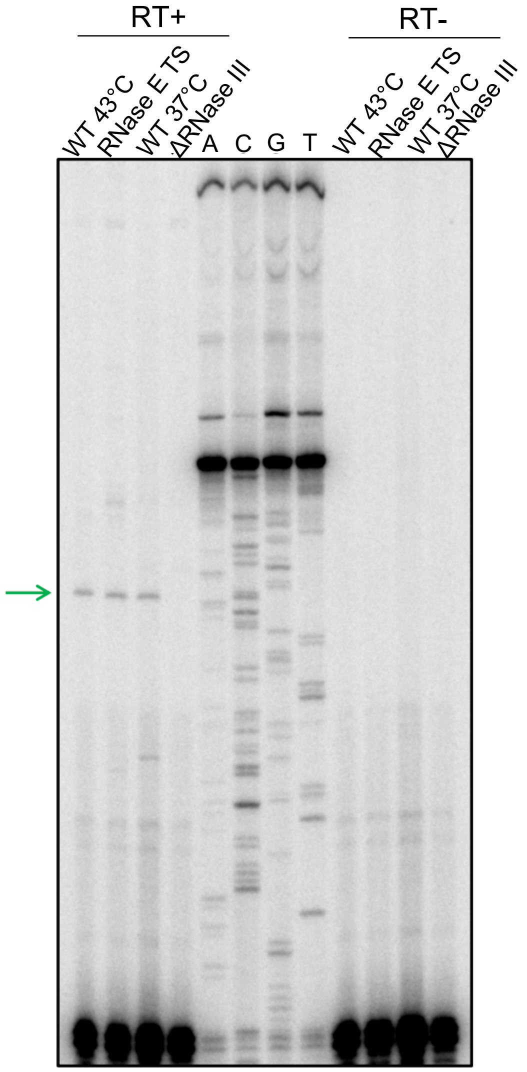

B

5′...gguagcugguucauagauagccugucguuaaauuuucgucgaccgugcgcuuccg**g**
uuguggcaacccgcgaaauggcgcggcgguaaguauggcgggguuauuccuuccccguu
gaggacaccggguugucagguugaccauacgcuuaagugacaaccccgcugcaacgccc
ucuguuaucaau**uuucuggugacguuuggcgguaucaguuuuacuccgugacugcucug**
ccgccc...3′

Figure S1. 5' end of the 190-nt DicF fragment is generated by RNase III processing of the *dicBF* transcript. A) Primer extension using radiolabeled *dicF* probe. The green arrow shows the RNase III cleavage site. B) Letter in green corresponds to the RNase III cleavage site on the DicF RNA sequence that was mapped on the primer extension in A. The 53-nt short version of DicF is underlined and in bold letters.

A

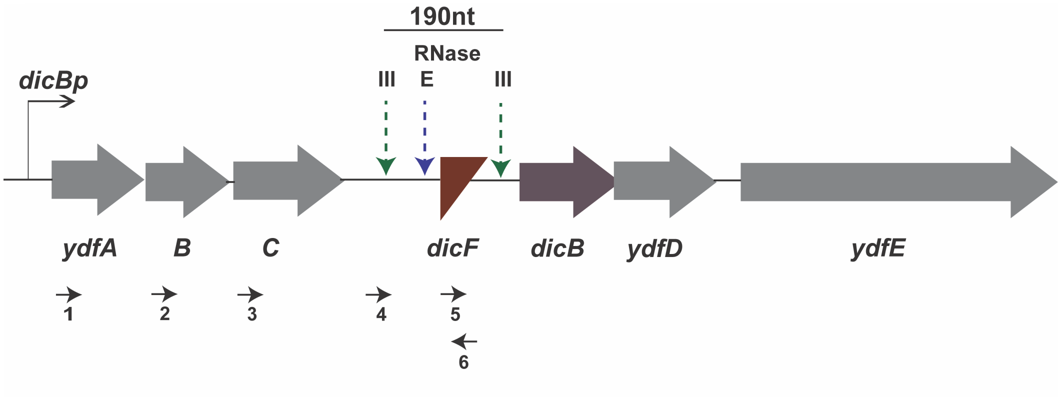

B

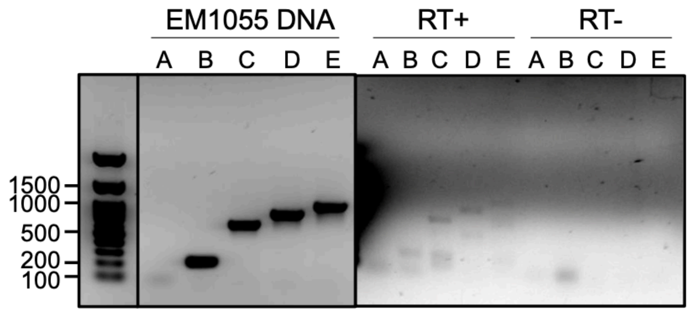

Lane	Primers	Product size
A	5+6	43 bp
B	4+6	206 bp
C	3+6	602 bp
D	2+6	760 bp
E	1+6	916 bp

Figure S2. DicF is processed from the transcript starting at *dicBp*. A) Schematic representation of primers used for RT- PCR on *ydfA* to *dicF* transcript. (B) RT-PCR on *ydfA* to *dicF* transcript was carried out on RNA extracted from WT *E. coli* K12 MG1655 cells grown aerobically in M63 minimal medium supplemented with glucose and 1μM $FeSO_4$. Genomic DNA of WT strain EM1055 was used as control.

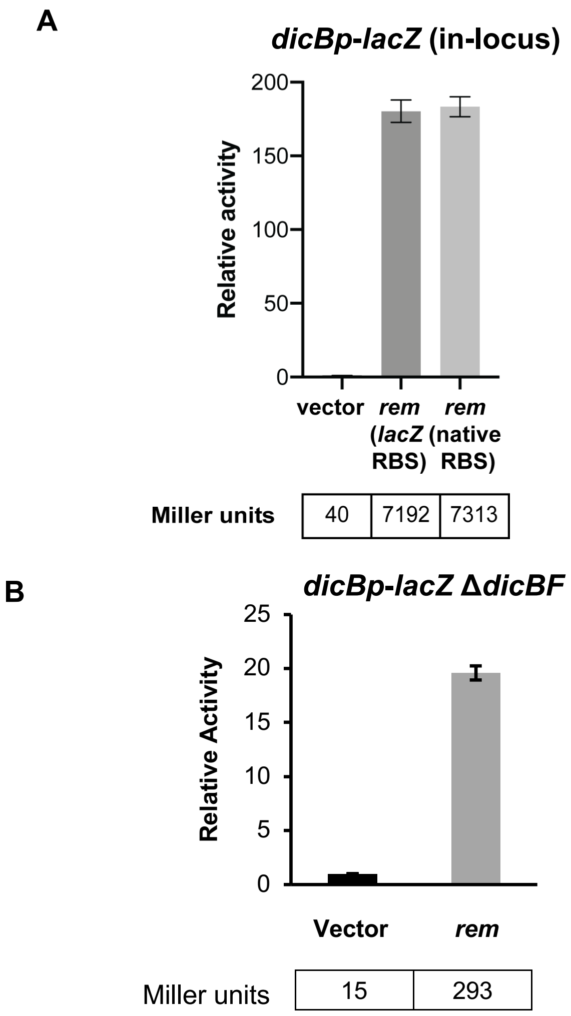

Figure S3. Rem is the antirepressor of *dicBp*. A) Miller assay was carried out with a *dicBp-lacZ* in-locus transcriptional fusion strain harboring either P_{tet}-vector, P_{tet}-*rem* having *lacZ* RBS, or P_{tet}-*rem* with its native RBS. The genes were induced for three hours with 10 ng/ml anhydrous tetracycline and β-galactosidase activity was assayed B) PR221 (*dicBp-lacZ* Δ*dicBF*) harboring P_{lac}-vector and P_{lac}-*rem* was grown until early log phase, induced with 0.1 mM IPTG for one hour, and β-galactosidase activity was assayed. The relative activity was calculated by dividing the Miller units of the specific strain to that of vector control. Error bars were calculated as standard deviation from three biological replicates.

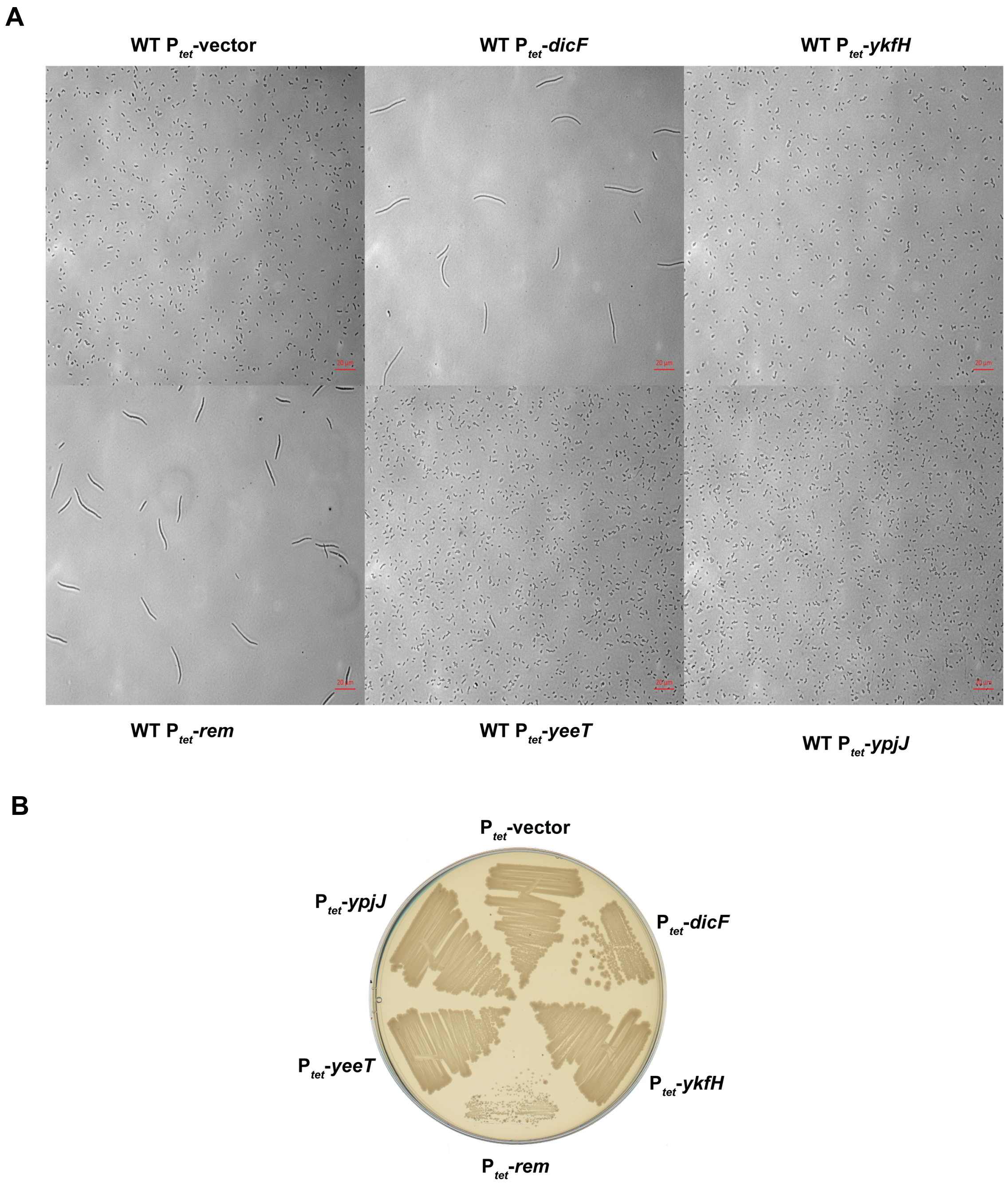

Figure S4. Rem is the only predicted antirepressors to cause filamentation and growth inhibition of cells. A) WT strain harboring P_{tet}-vector, P_{tet}-*dicF*, P_{tet}-*ykfH*, P_{tet}-*rem*, P_{tet}-*yeeT* or P_{tet}-*ypjJ* were grown for three hours in LB medium with 100 ng/ml anhydrous tetracycline and imaged using a bright field microscope. P_{tet}-*dicF* was used as a positive control for filamentation as the sRNA DicF is known to induce filamentation (13). B) The strains from A were streaked on LB agar plates with 100 ng/ml anhydrous tetracycline and incubated overnight at 37°C. P_{tet}-*dicF* was used as a positive control for growth inhibition as prolonged expression of DicF is toxic to the cells (13).

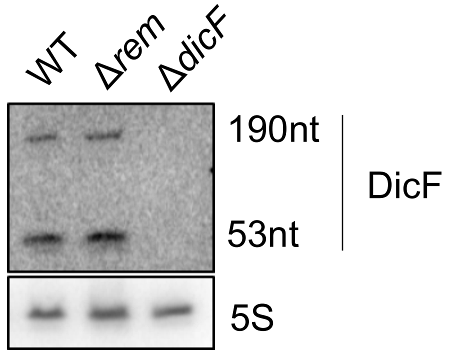

Figure S5. DicF expression during growth in M63 minimal media is independent of Rem. Northern blot showing the expression of DicF in a *rem* mutant strain. RNA was extracted at OD_{600} of 1.7-1.8 in M63 minimal media supplemented with 0.2% glucose and 1μM FeSO4. 5S RNA was used as a loading control.

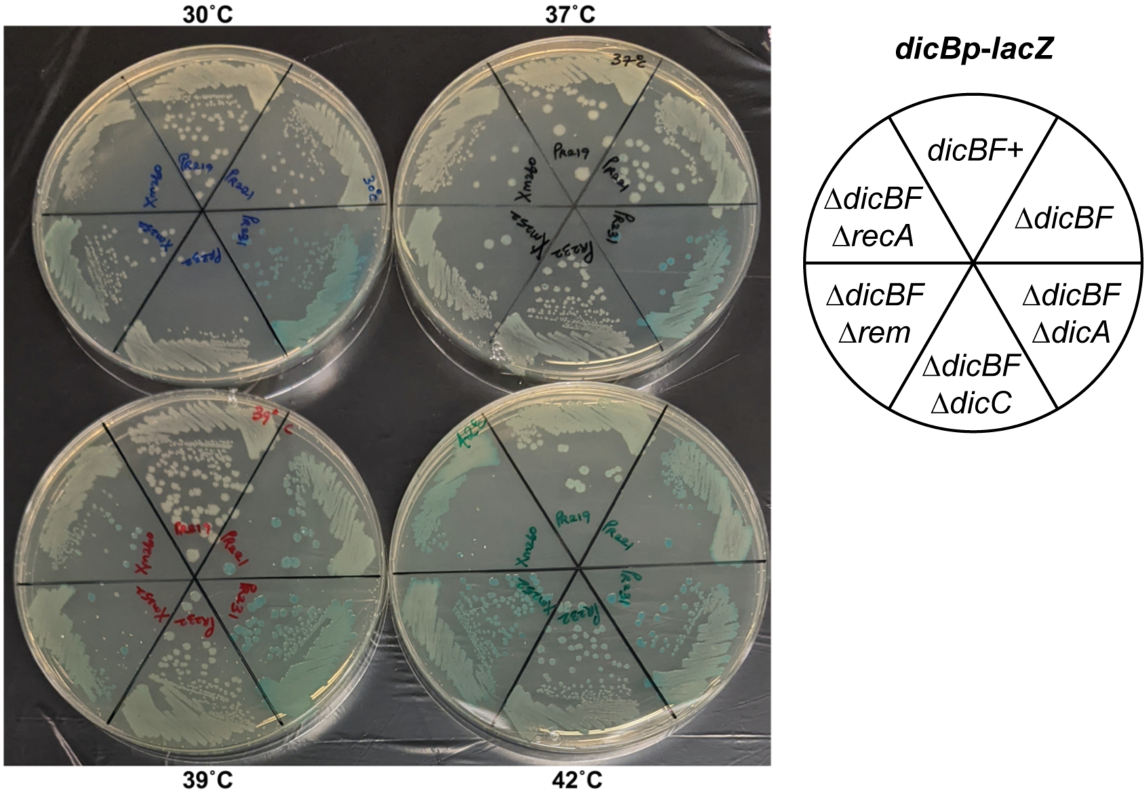

Figure S6. *dicBp* is induced by high temperature. The indicated strains were streaked on LB agar plates with X-Gal (40 µg/ml) and incubated overnight at 30, 37, 39, or 42°C.

Table S1. Strains and plasmids used in this study.

Strain name	Genotype	Source
DB166	DJ480 *lacIq* TetR SpecR	This study
DB181	DJ480 *lacIq* TetR SpecR Δ*dsrA*::tet	This study
EM1055	DJ480 (MG1655 Δ*lacX174)*	(44)
EM1277	EM1055 *rne-3071 zce-726*::Tn*10*	(24)
EM1321	EM1055 *rnc-14*::Tn*10*	(24)
ENG63	EM1055 Δ*dicF*::kan	This study
ENG262	EM1055 Δ*rem*::cat	This study
PM1805	DJ480 P_{BAD}::*cat-sacB*::*lacZ*, mini λTet	(43)
PR136	DB166 *dicBp-lacZ* (in-locus)	This study
PR142	DB166 Δ*qin*::*kan*	This study
PR143	DB166 Δ*dicB*::*kan*	This study
PR144	DB166 Δ*dicF*::*kan*	This study
PR145	DB166 Δ*qin*::scar	This study
PR146	DB166 Δ*dicB*::scar	This study
PR147	DB166 Δ*dicF* ::scar	This study
PR148	PR147 Δ*dicB*::scar	This study
PR149	DB166 Δ*dicF* Δ*dicB*	This study
PR219	PM1805 *dicBp-lacZ*	This study
PR221	PM1805 *dicBp-lacZ* Δ(*ydfA-intQ*)::tet	This study
PR226	PR221 Δ*dicA*::cm	This study
PR227	PR221 Δ*dicC*::cm	This study
PR231	PR221 Δ*dicA*::scar	This study
PR232	PR221 Δ*dicC*::scar	This study
XM252	PR221 Δ*rem*::scar	This study
XM260	PR221 Δ*recA*::scar	This study

Plasmid	Vector	Genotype	Source
pZA31R		Vector control	(45)
pZAPR1	pZA31R	P_{tet}-*dicF*	This study
pZAPR2	pZA31R	P_{tet}-*ypjJ*	This study
pZAPR3	pZA31R	P_{tet}-*ykfH*	This study
pZAPR4	pZA31R	P_{tet}-*yeeT*	This study
pZAPR5	pZA31R	P_{tet}-*rem*	This study
pZAPR6	pZA31R	P_{tet}-*rem* (native RBS)	This study
pBRPR7	pBRCS12	P_{lac}-*rem*	This study

Table S2. Oligonucleotides used in this study.

Oligo name	Description	Sequence (5'-3')
EM2267	Reverse primer for 16S DNA probe	CTGAAAGTACTTTACAACCCGAAGGCCTTCTTCATACACG
EM2268	Reverse primer for 5S DNA probe	CACACTACCATCGGCGCTACGGCGTTTCACTTCTGAGTTC
EM2655	Forward primer for *dicB* primer extension DNA matrix	CTGGTGACGTTTGGCGGTATC
EM2684	Reverse primer for *dicF* DNA probe	GAGCAGTCACGGAGTAAAACTGATACCGCCAAACGTCACC
EM2782	Forward primer for deleting *dicF* in EM1055	ACCATACGCTTAAGTGACAACCCCGCTGCAACGCCCTCTG GTGTAGGCTGGAGCTGCTTC
EM2783	Reverse primer for deleting *dicF* in EM1055	GCGTGCGCTCAGCCGCATTCACCACATCACAAAATTCACCA TATGAATATCCTCCTTAG
EM2784	Forward primer for RT-PCR between *ydfC* and *dicF* and *dicF* primer extension DNA matrix	GTCGTTAAATTTTCGTCGACCGTG
EM4668	Reverse primer for *dicF* primer extension DNA matrix	CATCCTCCCAATTTCAAAACAACCTTCAGACGTATTAAC
EM4721	Forward primer for RT-PCR in *dicF*	TTTCTGGTGACGTTTGGCGG
EM4722	Reverse primer for reverse transcription for RT-PCR experiments	GTGACTGCTCTGCCGCCC
EM4723	Forward primer for *ydfA* RNA probe	ATGGATACTATCGATCTTGGCAAC
EM4724	Reverse primer for *ydfA* RNA probe	TAATACGACTCACTATAGGGAGATCACTCACCTGAGTTTCTT TCC
EM4726	Reverse primer for *dicB* primer extension DNA matrix	CACATCCTTTTGGCATCAGAC
EM4753	Reverse primer for *dicF* primer extension probe	GTCACTTAAGCGTATGGTCAAC
EM5215	Forward primer for RT-PCR in *ydfB*	ATGGATTTCGACACAATCATGG
EM5216	Forward primer for RT-PCR in *ydfC*	ATGCAGAAACGAGAACCCGTC
EM5217	Reverse primer for RT-PCR	TATCAGTTTTACTCCGTGACTGC

EM5335	Reverse primer for *dicB* primer extension probe	CTTCAGTAAATACTGGGTTAC
O-PR124	Forward primer for Cm insertion at *dicBp*	CACTTGACCGATATGTTAGTCATGGCTAATCTTGTTTGCATGTAGGCTGGAGCTG
O-PR125	Reverse primer for Cm insertion at *dicBp*	GCCAAGATCGATAGTATCCATGCTGACCTCATTTCCCCTTCATATGAATATCCTCCTTAG
O-PR147	Forward primer for P_{tet}-*dicF* with Nde1 and BamH1 sticky ends	TATGTTTCTGGTGACGTTTGGCGGTATCAGTTTTACTCCGTGACTGCTCTGCCGCCCTTTTTG
O-PR148	Reverse primer for P_{tet}-*dicF* with Nde1 and BamH1 sticky ends	GATCCAAAAAGGGCGGCAGAGCAGTCACGGAGTAAAACTGATACCGCCAAACGTCACCAGAAACA
O-PR153	Forward primer for cloning *ypjJ* in pZA31R with NdeI site	CCCCCCCCCATATGTCACACAGGAAACAGCTATGAGAATTATCAGTAAACGCCGGGCAAT
O-PR154	Reverse primer for cloning *ypjJ* in pZA31R with BamHI site	CCCCCCCCGGATCCTCAGTCAAGCATACGGTCCGCT
O-PR155	Forward primer for cloning *yeeT* in pZA31R with NdeI site	CCCCCCCCCATATGTCACACAGGAAACAGCTATGAAAATTATCACCCGTGGTGAAGCCAT
O-PR156	Reverse primer for cloning *yeeT* in pZA31R with BamHI site	CCCCCCCCGGATCCTCAGTTCAGGGTGACGCTCATCAG
O-PR157	Forward primer for cloning *ykfH* in pZA31R with NdeI site	CCCCCCCCCATATGTCACACAGGAAACAGCTATGAAAATTATCAGTAAACGCAGGGCAAT
O-PR158	Reverse primer for cloning *ykfH* in pZA31R with BamHI site	CCCCCCCCGGATCCTCAGTTCAGGGTGATGCTCATC
O-PR159	Forward primer for cloning *rem* in pZA31R with NdeI site	CCCCCCCCCATATGTCACACAGGAAACAGCTATGATGAACATCGAAGAACTGCGTAAAAT
O-PR160	Reverse primer for cloning *rem* in pZA31R with BamHI site	CCCCCCCCGGATCCTTATTCTCCTGAAAAAGCAGAAAAGCCA
O-PR163	Forward primer for cloning *rem* with native RBS in pZA31R with NdeI site	CCCCCCCCCATATGATCCGGAAGGATTCTGATGATGAACAT

O-PR164	Reverse primer for cloning *rem* with native RBS in pZA31R with BamHI site	CCCCCCCCGGATCCTTATTCTCCTGAAAAAGCAGAAAAGCCA
O-PR230	Forward primer to delete *ydfA*-*intQ* with tet	TCTCTGGTGTACGGCGTGTTTCCAAACCAGGACGGCACGTCTAGACATCATTAATTCCTAATTTTTGTTGACA
O-PR231	Reverse primer to delete *ydfA*-*intQ* with tet	TTCAAGAGTCATGCGTGTTAACTATTTGATAAATATTAAAAGGTTTTATTTGAAGCTAAATCTTCTTTAT
O-PR241	Forward primer for *dicBp*-*lacZ* transcriptional fusion in PM1805	CTATGCCATAGCATTTTTATCCATAAGATTAGCGGATCCCATCATCAATGAGTTATCTT
O-PR242	Reverse primer for *dicBp*-*lacZ* transcriptional fusion in PM1805	TAACGCCAGGGTTTTCCCAGTCACGACGTTGTAAAACGACCATAGCTGTTTCCTGTGTGACCGTGCGGTGTGTTGATGCA
O-PR286	Forward primer for cloning *rem* in pBRCS12 with BamH1 restriction site	CCCCCCCCGGATCCTCACACAGGAAACAGCTATGATGAACATCGAAGAACTGCG
O-PR287	Reverse primer for cloning *rem* in pBRCS12 with HindIII restriction site	CCCCCCCCAAGCTTTTATTCTCCTGAAAAAGCAGAAAAGCC
O-PR299	Forward primer for deleting *dicA* in PR221	TAACAAAAACATAGTCAATACGATTTAGCATTAGCTAACTTGTAGGCTGGAGCTG
O-PR300	Reverse primer for deleting *dicA* in PR221	TAACATAATTGATGTGGTAAAAGATAACTCATTGATGATGCATATGAATATCCTCCTTAG
O-PR303	Forward primer for deleting *dicC* in PR221	TCTATCTTGTTAGTTATGACTAACAATAAAGGTGTTTTAATGTAGGCTGGAGCTG
O-PR304	Reverse primer for deleting *dicC* in PR221	TTTCATTGTTCAACCGCCCCGCCCGCTTCGTCTTACGATACATATGAATATCCTCCTTAG
O-PR307	Forward primer for deleting *rem* in PR221	GTCCCTTAAAGGGGAGAGCTAATTATCCGGAAGGATTCTGTGTAGGCTGGAGCTG
O-PR308	Reverse primer for deleting *rem* in PR221	CTCAGCGGGAGTTCAGCCCCGCGCAAGATTGTAGATGAGTCATATGAATATCCTCCTTAG
O-PR311	Forward primer for deleting *recA* in PR221	TATTGACTATCCGGTATTACCCGGCATGACAGGAGTAAAATGTAGGCTGGAGCTG
O-PR312	Reverse primer for deleting *recA* in PR221	AAGGGCCGCAGATGCGACCCTTGTGTATCAAACAAGACGACATATGAATATCCTCCTTAG
